# DNA methylation of exercise-responsive genes differs between trained and untrained men

**DOI:** 10.1186/s12915-024-01938-6

**Published:** 2024-07-04

**Authors:** Carla Geiger, Maria Needhamsen, Eric B. Emanuelsson, Jessica Norrbom, Karen Steindorf, Carl Johan Sundberg, Stefan M. Reitzner, Malene E. Lindholm

**Affiliations:** 1 Department of Physiology and Pharmacology, Karolinska Institutet, Stockholm, Sweden; 2 Division of Physical Activity, Prevention and Cancer, German Cancer Research Center (DKFZ) and National Center for Tumor Diseases (NCT), Heidelberg, Germany; 3 Medical School, Heidelberg University, Heidelberg, Germany; 4 Department of Clinical Neuroscience, Center for Molecular Medicine, Karolinska Institutet, Karolinska University Hospital, Stockholm, Sweden; 5 Department of Learning, Informatics, Management and Ethics, Karolinska Institutet, Stockholm, Sweden; 6 Department of Laboratory Medicine, Karolinska Institutet, Huddinge, Sweden; 7 Department for Women’s and Children’s Health, Karolinska Institutet, Stockholm, Sweden; 8 Center for Inherited Cardiovascular Disease, School of Medicine, Stanford University, 870 Quarry Rd, Stanford, CA 94305 USA

**Keywords:** DNA methylation, Epigenomics, Enzymatic methyl sequencing, Exercise, Training, Gene expression

## Abstract

**Background:**

Physical activity is well known for its multiple health benefits and although the knowledge of the underlying molecular mechanisms is increasing, our understanding of the role of epigenetics in long-term training adaptation remains incomplete. In this intervention study, we included individuals with a history of > 15 years of regular endurance or resistance training compared to age-matched untrained controls performing endurance or resistance exercise. We examined skeletal muscle DNA methylation of genes involved in key adaptation processes, including myogenesis, gene regulation, angiogenesis and metabolism.

**Results:**

A greater number of differentially methylated regions and differentially expressed genes were identified when comparing the endurance group with the control group than in the comparison between the strength group and the control group at baseline. Although the cellular composition of skeletal muscle samples was generally consistent across groups, variations were observed in the distribution of muscle fiber types. Slow-twitch fiber type genes *MYH7* and *MYL3* exhibited lower promoter methylation and elevated expression in endurance-trained athletes, while the same group showed higher methylation in transcription factors such as *FOXO3*, *CREB5*, and *PGC-1α*. The baseline DNA methylation state of those genes was associated with the transcriptional response to an acute bout of exercise. Acute exercise altered very few of the investigated CpG sites.

**Conclusions:**

Endurance- compared to resistance-trained athletes and untrained individuals demonstrated a different DNA methylation signature of selected skeletal muscle genes, which may influence transcriptional dynamics following a bout of acute exercise. Skeletal muscle fiber type distribution is associated with methylation of fiber type specific genes. Our results suggest that the baseline DNA methylation landscape in skeletal muscle influences the transcription of regulatory genes in response to an acute exercise bout.

**Supplementary Information:**

The online version contains supplementary material available at 10.1186/s12915-024-01938-6.

## Background

Regular physical activity has significant health benefits, including improved cardiovascular health, metabolic function, and overall well-being [1]. Changes in gene expression in skeletal muscle are pivotal in driving exercise adaptations [2–5]. In fact, a single episode of acute exercise can induce transient alterations in the skeletal muscle transcriptome [4, 6–9]. Moreover, there is a notable difference in gene expression between trained and untrained skeletal muscles even at rest [2, 10]. This is particularly evident in endurance-trained individuals [2, 11].

Gene activity is regulated by multiple mechanisms including epigenetics, such as DNA methylation [12]. Typically, methylation of promoter regions is associated with transcriptional suppression, whereas the effects of intra- and intergenic methylation on gene transcription are variable [12, 13]. Historically, DNA methylation patterns were perceived as stable modifications, primarily established during early development, and only changing over extended periods, as seen during aging and in chronic disease [14–16]. However, a growing body of evidence indicates that various environmental factors can induce more immediate modifications in DNA methylation levels [17–20]. Importantly, it has been demonstrated that physical activity can impact DNA methylation, thereby potentially playing a role in facilitating physiological adaptations to exercise and, consequently, exercise-induced health benefits [21–25]. Only a handful of studies have explored epigenetic patterns that occur in a trained state and the immediate alterations in DNA methylation following exercise. These studies suggest a role for epigenetic mechanisms in skeletal muscle memory by preserving molecular information within cells following environmental stimuli, thereby creating a primed state for subsequent stimuli [13, 26–28]. Evidence points to an association between dynamic DNA methylation changes and acute gene activation following exercise [29]. However, our understanding of epigenetic regulation in response to exercise remains incomplete [30].

Here, we investigated whether the DNA methylation patterns of exercise responsive genes are modified by training status or the response to an acute exercise bout in individuals with variable training backgrounds. Our targeted panel focused on key transcription factor genes like forkhead box O (*FOXOs*), myogenic regulatory factors (MRFs), and cAMP response element-binding protein (*CREB*), which are important for skeletal muscle mass and function regulation [3]. Members of the *FOXO* protein family regulate processes such as cell cycle, apoptosis, and muscle development and repair [31], where *FOXO1* and *FOXO3* are pivotal in maintaining muscle energy homeostasis [31, 32]. MRFs are key transcription factors involved in the regulation of muscle development and adaptation [33, 34]. The MRF family includes *MYF5*, *MYOD1*, *MYOG*, and *MYF6* (*MRF4*), all of which play crucial roles in muscle cell differentiation and growth. *MEF2A* (myocyte enhancer factor 2) is another important transcription factor that interacts with the MRFs to regulate muscle gene expression [35]. The *CREB* protein family plays an important role in various cellular processes, including the regulation of gene expression, which in turn influences hypertrophic growth, metabolic efficiency, and muscle performance [36]. The panel also included different regions of peroxisome proliferator-activated receptor gamma coactivator 1-alpha (*PGC-1α*), a transcriptional coactivator that mediates exercise-induced health benefits by regulating genes involved in energy metabolism, mitochondrial biogenesis, angiogenesis, fiber-type switching, and neuromuscular junction remodelling [37, 38]. In addition, genes specific to muscle fiber types were also part of the selection. In skeletal muscle, the expression of specific myosin heavy and light chain proteins dictates the contractile characteristics of muscle fibers. Type I (slow-twitch) fibers predominantly contain *MYH7*, *MYH6*, and *MYL3* proteins. The presence of *MYH6* and *MYH7* indicates a slow-twitch phenotype, while their absence defines a fast-twitch phenotype [39]. Fast type IIa fibers are marked by the presence of *MYH2* [40].

Understanding the DNA methylation status of exercise-responsive genes in subjects with distinctly different training backgrounds and physiological phenotypes can provide insights into the molecular mechanisms underlying exercise-induced adaptations in skeletal muscle. We therefore aimed to investigate DNA methylation and associated gene expression of selected genes in trained versus untrained skeletal muscle at baseline and after one bout of endurance or resistance exercise.

## Results

### Characterisation of study participants and analyses performed

**Fig. 1:**
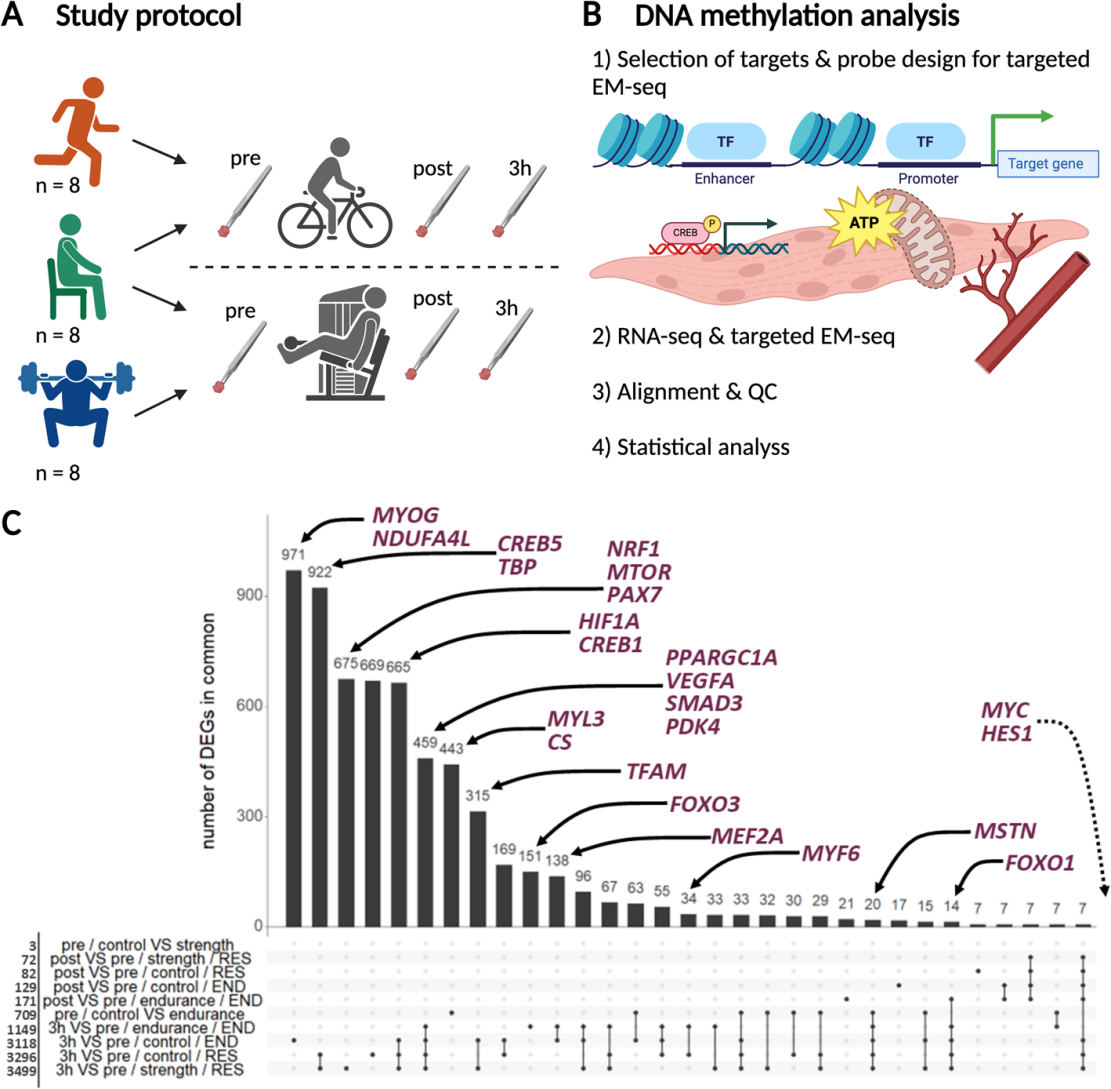
Study design and gene expression analysis. **A** Overview of the study design. Endurance-trained men (orange), untrained healthy men (controls, green) and strength-trained men (blue) performed acute endurance and/or resistance exercise. Skeletal muscle was collected at three timepoints; pre = before exercise, post = immediately after exercise and at 3 h after exercise. **B** Gene expression and targeted DNA methylation of regulatory regions of selected genes with known function in skeletal muscle adaptation to exercise were analysed. **C** UpSet plot of differentially expressed genes (DEGs) for all group comparisons. Genes analysed for DNA methylation are labelled if among the DEGs. END = acute endurance exercise, RES = acute resistance exercise. For visualisation purposes, only differential gene comparisons down to 7 genes are shown. MYC and HES were also differentially expressed (see Additional file 1: Table S1 for all panel genes). The complete list of all genes can be found in Additional file 2. Created with BioRender.com

Twenty-four healthy men (mean age of 41.6 years ± 6.0 SD) were recruited based on training background and divided into three distinct groups, as previously reported [11]. The endurance group (EG, *n* = 8) and strength group (SG, *n* = 8) included lifelong high-level endurance- and strength-trained athletes respectively, which were compared to an age-matched untrained control group (CG, *n* = 8). Participants were assigned to their respective groups based on self-reported training history, physiological testing of their VO_2_ peak, and peak knee extension torque [11]. Endurance-trained athletes exhibited a significantly higher VO_2_ peak (67.0 ± 7.2 ml/min/kg) compared to the other groups (CG: 36.2 ± 4.4 ml/min/kg; SG: 40.2 ± 6.9 ml/min/kg) and higher skeletal muscle citrate synthase activity [11], an indicator of mitochondrial content [41–43]. The endurance group also had a significantly higher proportion of slow-twitch type I fibers [11]. Conversely, strength-trained athletes displayed significantly greater leg strength (289.4 ± 28.1 Nm) compared to individuals in the CG (180.6 ± 30.8 Nm) and EG (198.3 ± 25.5 Nm). Additionally, the cross-sectional area of their fast-twitch type II fibers was substantially larger than that observed in the other groups [11]. All participants performed acute exercise with skeletal muscle biopsies collected from *m. vastus lateralis* at three different timepoints (Fig. 1A, the ‘Methods’ section).

To interrogate changes in DNA methylation, we employed enzymatic methyl sequencing (EM-seq) of selected genes involved in key aspects of exercise adaptation such as myogenesis, gene regulation, angiogenesis, and metabolism and/or shown to be differentially expressed between groups at baseline or in response to exercise (Fig. 1B, C). The targeted panel included probes spanning the promoter regions, including transcriptional start sites, as well as enhancer-associated elements of genes. We selected transcription factors such as *CREBs*, *FOXOs* and the myogenic regulatory factors, genes that are specific to fiber types, and genes contributing to cell growth, protein synthesis pathways, hypoxia response, angiogenesis, mitochondrial biogenesis, and energy regulation and metabolism. In total, 57 target regions across 37 genes were selected, covering 64,969 bp (Additional file 1: Table S1). Processing of the resulting EM-seq data was performed using the nf-core/methylseq pipeline [44]. Base calls were of good quality with high coverage of the target regions (the ‘Methods’ section).

Transcriptomic analysis of the skeletal muscle tissue at baseline identified 709 differentially expressed genes (DEGs) between the control and endurance groups and only 3 DEGs between the control and strength groups (Fig. 1C, Additional file 2). Next, to investigate potential regulators driving the differential gene expression observed between groups, we conducted an epigenetic Landscape In Silico deletion Analysis (Lisa) [45]. The top 25 predicted regulators of higher gene expression in the EG included *MYOD1*, *MYC*, and *MYOG*, known regulators of exercise-induced gene expression (Additional file 3). We were specifically interested in putative regulators of DNA methylation differences between groups and identified the known methylation regulating enzymes *TET2*, *TET3*, *DNMT3A*, and *DNMT3* among the significant predictive regulators of lower gene expression in the endurance group.

Gene expression and DNA methylation analysed at the bulk tissue level is affected by the sample cell type composition. To estimate cell type composition of the muscle samples, we interrogated the transcriptome data for specific marker genes of endothelial cells (*VWF*, *ESAM*, *KDR*, *ICAM1*, *PECAM-1/CD31*, and *CD144*), mesenchymal cells (*DCN*, *CFD*, and *GSN*), myogenic cells (*PAX7*), and interstitial fibro-adipogenic progenitors (*TEK*) [11, 46, 47]. No differences in expression between groups were observed at baseline. Immune cell type composition, analysed using CIBERSORTx [48], was largely similar between groups, with only slightly higher resting NK cells in the endurance group compared to controls (FDR < 0.01) and lower M2 macrophages (FDR = 0.04) comparing the endurance to the strength group (Additional file 1: Table S2).

### *Fiber*-type specific and regulatory genes are differentially methylated in endurance-trained skeletal muscle

**Fig. 2:**
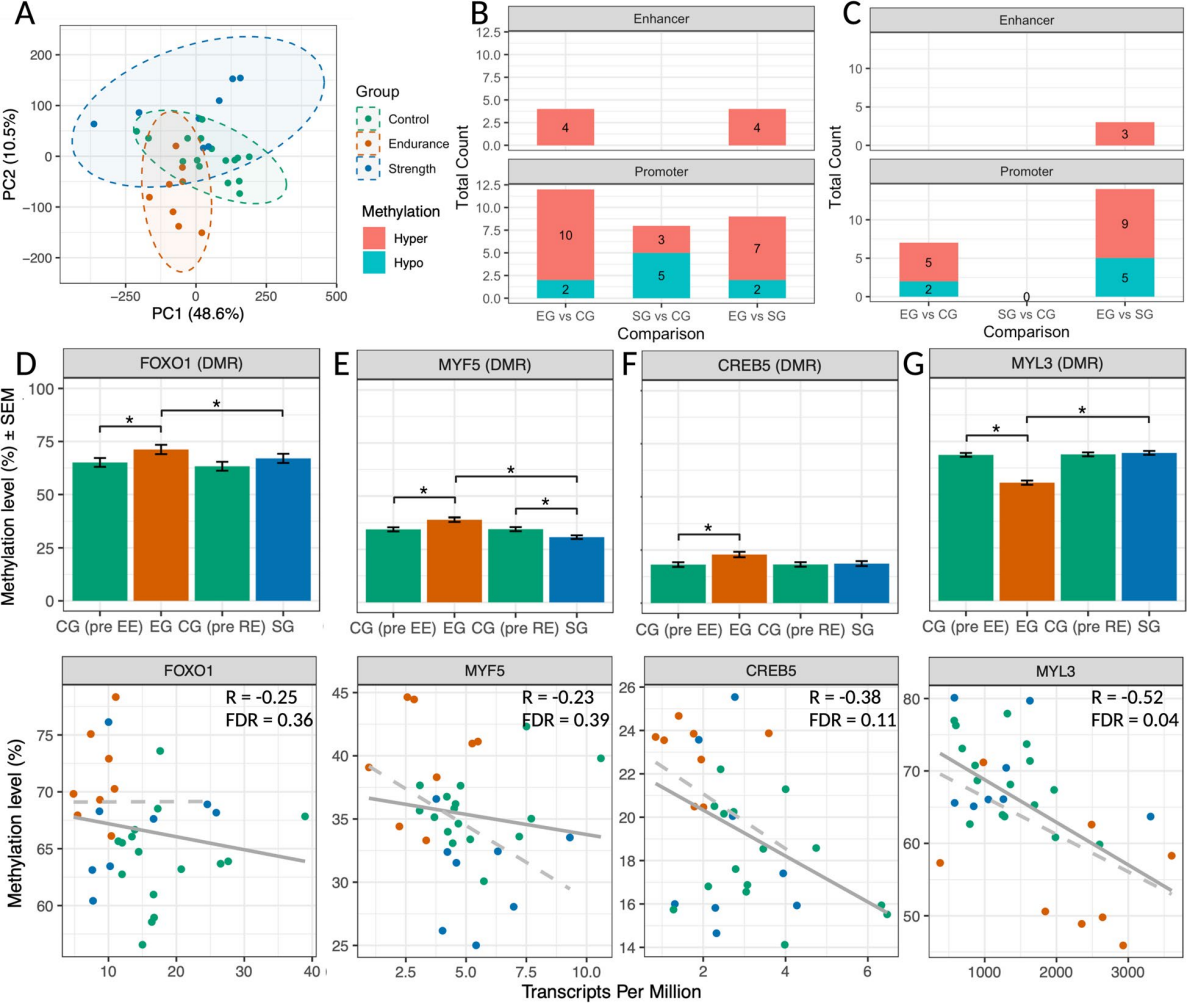
Baseline skeletal muscle DNA methylation differences. **A** Unsupervised principal component analysis using methylation percentage of all CpG sites with 10X coverage across the target probes. Samples are coloured by group (green = control group/CG, orange = endurance group/EG, blue = strength group/SG). The dashed ellipses represent the 95% confidence regions for the multivariate mean of each group in the PC1 vs PC2 space, providing a visual representation of the dispersion and overlap of the groups. See Additional file 6 for individual methylation percentages. **B** Number of differentially methylated regions (DMRs) and **C** number of differentially methylated positions (DMPs) comparing the 3 groups at rest. Red indicates higher methylation and blue indicates lower methylation. See Additional file 4 for all DMRs and DMPs. **D**–**G** Top: bar plots of selected DMRs at baseline comparing EG, SG, and CG. Overlapping DMRs from different group comparisons are plotted in the same figure. Results are shown as mean ± SEM for *n* = 8 subjects for FOXO1 (**D**), MYF5 (**E**), CREB5 (**F**), and MYL3 (**G**). ∗ indicates *p* < 0.05. Bottom: Scatter plots showing correlation between mean methylation of DMRs and gene expression of the associated gene for all samples. *R* = Spearman correlation coefficient. Full line = linear regression model including subjects from all groups. Dotted line = linear regression model only including trained individuals (SG and EG). *Y*-axes vary for better visualisation of the regression lines. See Additional file 6 for individual methylation percentages and TPM values

First, the potential influence of training background on DNA methylation of exercise-responsive genes was investigated. In an unsupervised principal component (PC) analysis, individuals from the same group largely clustered together in both PC1 and PC2, which accounted for 49% and 10% of the variance, respectively (Fig. 2A). Notably, the endurance-trained group appeared to diverge from both the strength-trained and the untrained control groups. A Kruskal–Wallis test confirmed significant differences between the groups for both PC1 (*p* < 0.01) and PC2 (*p* < 0.001). The specific differential methylation analysis identified 16 differentially methylated regions (DMRs, Fig. 2B) and 7 differentially methylated positions (DMPs, Fig. 2C) in the endurance athletes compared to controls at baseline. Fewer differences were identified between the strength and control groups: 8 DMRs (*p* < 0.05) and no individual DMPs. The endurance athletes exhibited higher methylation in the majority of DMRs compared to the other groups (Table 1). Intronic DMRs for the central transcription factor genes *FOXO1* and *FOXO3* were more methylated in the endurance athletes, although there was no difference in baseline gene expression between the groups (Table 1, Fig. 2D, Additional file 2). Methylation levels of *FOXO3* and *FOXO1* also positively correlated to VO_2_ peak (Additional file 1: Table S3). Greater methylation in the EG was also observed in a *MYF5* promoter DMR compared to both the CG and the SG, with the SG having lower methylation levels than the CG (Table 1, Fig. 2E). We identified three DMRs around the *MYOG* promoter: the EG had higher methylation compared to the CG for one DMR, while the SG had higher methylation compared to the CG for the other two DMRs (Table 1). We did not identify any DMR for *MYF6*.

**Table 1:** Differentially methylated regions (DMRs) between groups at baseline

***DMRs: endurance group versus control group at baseline***							
**Gene**	**Associated feature**	**Length**	**nCpG**	**EG-DNA methylation**	**CG-DNA methylation**	**Methylation difference**	**areaStat**
*MTOR*	*Enhancer (intron)*	*95*	*6*	*66%*	*57%*	*9%*	*20*
*MYOG*	*Promoter*	*1104*	*46*	*36%*	*29%*	*7%*	*129*
*MYF5*	*Promoter*	*207*	*32*	*39%*	*34%*	*5%*	*67*
*FOXO1*	*Enhancer (intron)*	*729*	*14*	*76%*	*70%*	*7%*	*42*
*MYH7*	*Promoter*	*174*	*9*	*18%*	*23%*	*-5%*	*-24*
*SMAD3*	*Promoter*	*444*	*23*	*30%*	*22%*	*8%*	*95*
*SMAD3*	*Promoter*	*128*	*7*	*27%*	*22%*	*5%*	*16*
*MEF2A*	*Enhancer (intron)*	*191*	*6*	*58%*	*47%*	*11%*	*20*
*MYH2*	*Promoter*	*223*	*10*	*35%*	*29%*	*6%*	*28*
*MAPK12*	*Promoter*	*299*	*14*	*42%*	*34%*	*8%*	*51*
*MYL3*	*Promoter*	*475*	*14*	*56%*	*69%*	*-13%*	*-62*
*PPARGC1A*	*Proximal promoter (ex1a_a)*	*187*	*12*	*32%*	*25%*	*7%*	*36*
*PPARGC1A*	*Proximal promoter (ex1a)*	*271*	*9*	*12%*	*9%*	*3%*	*19*
*PPARGC1A*	*Alternative promoter (ex1b)*	*76*	*6*	*53%*	*42%*	*11%*	*27*
*FOXO3*	*Enhancer (intron)*	*1291*	*19*	*56%*	*50%*	*6%*	*53*
*CREB5*	*Promoter*	*101*	*7*	*26%*	*21%*	*5%*	*20*
***DMRs: strength group versus control group at baseline***							
**Gene**	**Associated feature**	**Length**	**nCpG**	**SG-DNA methylation**	**CG-DNA methylation**	**Methylation difference**	**areaStat**
*MYOG*	*Promoter*	*101*	*19*	*56%*	*46%*	*10%*	*54*
*MYOG*	*Promoter*	*67*	*7*	*51%*	*42%*	*9%*	*15*
*MYOD1*	*Promoter*	*57*	*7*	*16%*	*11%*	*5%*	*22*
*MYF5*	*Promoter*	*619*	*48*	*31%*	*37%*	*-6%*	*-134*
*HIF1A*	*Promoter*	*170*	*6*	*62%*	*69%*	*-7%*	*-17*
*SMAD3*	*Promoter*	*60*	*9*	*71%*	*75%*	*-4%*	*-15*
*SMAD3*	*Promoter*	*87*	*9*	*79%*	*84%*	*-5%*	*-18*
*CREB1*	*Promoter*	*77*	*6*	*14%*	*19%*	*-4%*	*-13*
***DMRs: endurance group versus strength group at baseline***							
**Gene**	**Associated feature**	**Length**	**nCpG**	**EG-DNA methylation**	**SG-DNA methylation**	**Methylation difference**	**areaStat**
*MTOR*	*Enhancer (intron)*	*95*	*6*	*66%*	*50%*	*16%*	*26*
*MYF5*	*Promoter*	*813*	*78*	*39%*	*30%*	*9%*	*319*
*FOXO1*	*Enhancer (intron)*	*91*	*6*	*86%*	*80%*	*5%*	*15*
*MYH7*	*Promoter*	*174*	*9*	*18%*	*22%*	*-4%*	*-21*
*SMAD3*	*Promoter*	*445*	*24*	*32%*	*24%*	*8%*	*97*
*SMAD3*	*Promoter*	*94*	*8*	*47%*	*39%*	*8%*	*19*
*SMAD3*	*Promoter*	*88*	*7*	*55%*	*48%*	*7%*	*17*
*SMAD3*	*Promoter*	*173*	*7*	*29%*	*24%*	*5%*	*16*
*CREB1*	*Promoter*	*77*	*6*	*19%*	*14%*	*4%*	*15*
*MAPK12*	*Enhancer*	*97*	*6*	*58%*	*53%*	*4%*	*13*
*MYL3*	*Promoter*	*475*	*14*	*56%*	*70%*	*-14%*	*-63*
*PPARGC1A*	*Proximal promoter (ex1a_a)*	*103*	*9*	*31%*	*26%*	*5%*	*18*
*FOXO3*	*Enhancer (intron)*	*117*	*6*	*78%*	*70%*	*7%*	*20*

Default parameters with a p-value threshold of < 0.05 were utilised to identify DMRs. areaStat = sum of the test statistics of all CpG sites within a DMR (larger areaStat is more likely to be a DMR). See Additional file 4 for genomic locations of DMRs as well as information on differentially methylated positions (DMPs). Individual data values can be found in Additional file 6

Strength athletes had higher *MYOD1* methylation compared to the CG (Table 1), and there was a positive correlation between degree of *MYOD1* methylation and leg strength across all individuals (Additional file 1: Table S3). No difference in baseline gene expression between the groups was identified for any of the MRFs (Additional file 2). For *MEF2A*, increased methylation of an intronic DMR was observed in endurance-trained athletes (Table 1). A DMR was detected near the promoter of the transcriptional regulator *CREB5*, with higher methylation in endurance athletes compared to the control group, which also positively correlated with VO_2_ peak across all three groups, (Table 1, Fig. 2F, Additional file 1: Table S3). Baseline gene expression showed a weak negative correlation between methylation and expression of *CREB5* (*R* =  − 0.38, FDR = 0.11, Fig. 2F). The SG exhibited lower methylation levels for a DMR in the *CREB1* promoter compared to both the CG and EG (Table 1), but there was no difference in gene expression or correlation with VO_2_ peak.

Endurance athletes had hypomethylated DMRs near the *MYH7* and the *MYL3* promoters compared to the other groups, which negatively correlated with VO_2_ peak (Table 1, Fig. 2G, Additional file 1: Table S3). Within the *MYL3* DMR, there were three hypomethylated DMPs (Additional file 4). Gene expression of *MYL3* was significantly higher in endurance athletes (Fig. 1B, Additional file 2), with a moderate negative correlation between *MYL3* expression and promoter methylation (*R* =  − 0.52, FDR = 0.04, Fig. 2G). Additionally, we observed that the methylation status of the *MYH7* and *MYL3* DMRs negatively correlated with the proportion of type I fibers (Additional file 1: Table S3) and positively with the proportion of type II fibers. In the case of *MYH2*, one DMR with higher methylation in the EG compared to the CG was found. *MYH2* expression did not differ significantly between the groups but the EG showed a trend towards lower expression levels (Additional file 2). *MYH2* methylation did not correlate with fiber type distribution.

### One bout of exercise alters few DNA methylation sites in selected genes

**Fig. 3:**
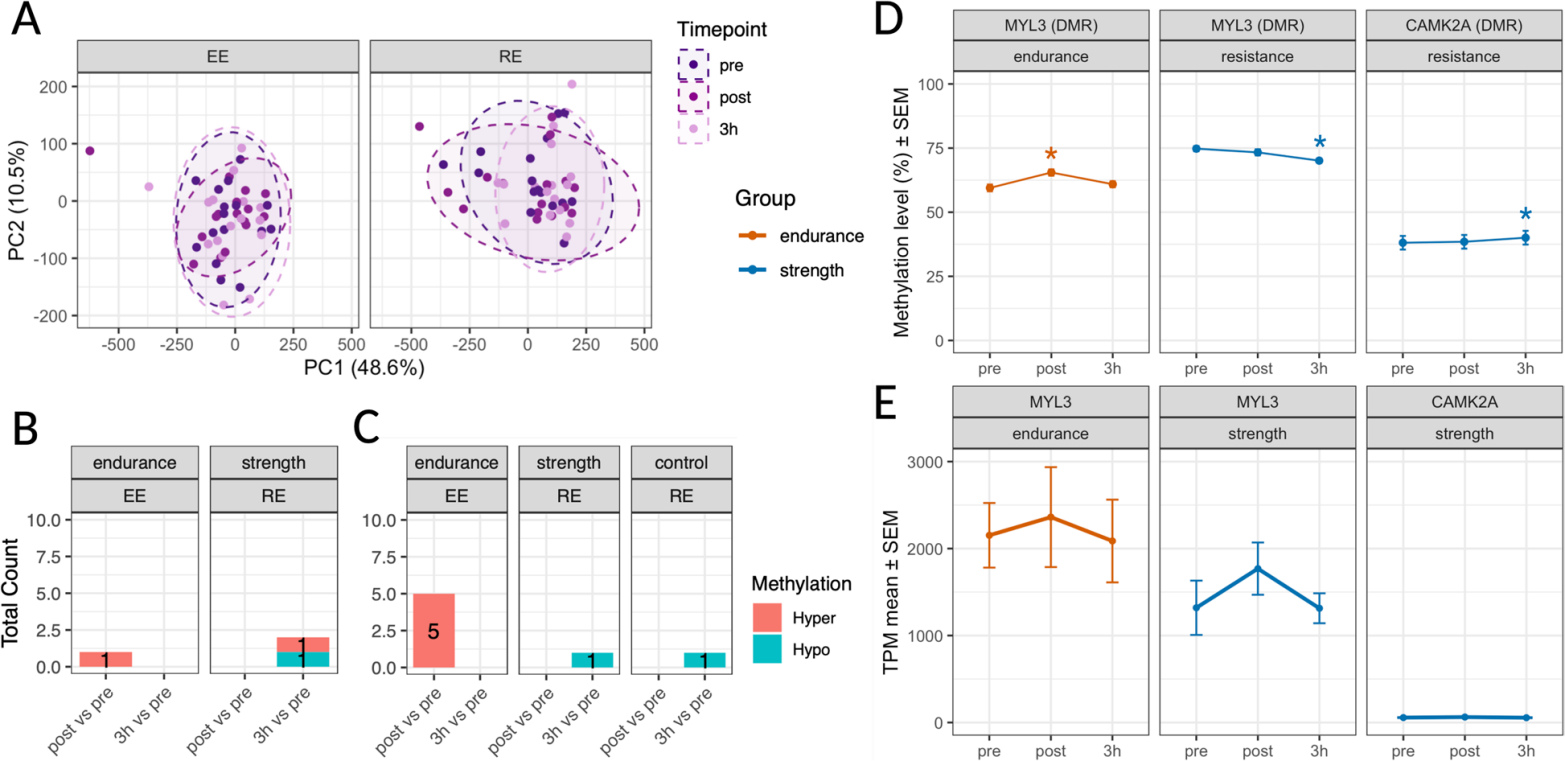
DNA methylation changes in response to exercise. **A** Principal component analysis using methylation percentage of all CpG sites. Samples are coloured by timepoint (dark purple = pre/before exercise, purple = post/immediately after exercise, rose = 3 h after exercise). The dashed ellipses represent the 95% confidence regions for the multivariate mean of each timepoint in the PC1 vs PC2 space. Left panel: EE = acute endurance exercise, right panel: RE = acute resistance exercise. See Additional file 6 for individual methylation percentages. **B** Number of differentially methylated regions (DMRs) and **C** number of differentially methylated positions (DMPs) immediately (post) and 3 h after exercise. No changes were observed in interventions/groups not depicted. EE = acute endurance exercise, RE = acute resistance exercise. Red indicates DMRs or DMPs with increased methylation (Hyper) and blue decreased methylation (Hypo) after exercise. See Additional file 5 for all DMRs and DMPs. **D** Significant DMRs in response to exercise. Results are displayed as mean ± SEM for *n* = 8 subjects. ∗ indicates *p* < 0.05. **E** Expression changes of genes associated to DMRs depicted in D. See Additional file 6 for individual methylation percentages and TPM values

To investigate whether DNA methylation exhibits dynamic changes in response to resistance or endurance exercise in the investigated groups, we analysed skeletal muscle DNA methylation immediately and 3 h after exercise for each group. PCA did not reveal any timepoint-specific separation (Fig. 3A), and few differentially methylated positions were identified in the selected regions (Fig. 3B and Fig. 3C, Additional file 5).

In endurance athletes performing endurance exercise, we observed an increase in DNA methylation within the *MYL3* promoter region (one DMR and five DMPs) immediately after exercise. Notably, strength-trained athletes exhibited a significant decrease in methylation within a DMR on the *MYL3* promoter 3 h after resistance exercise (Fig. 3D, Table 2). However, neither group showed exercise-induced transcriptional changes in the *MYL3* gene (Fig. 3E). The SG exhibited increased methylation of a DMR in the *CAMK2A* promoter (Fig. 3D, Table 2). In contrast, one DMP located farther from the transcription start site showed a decrease in methylation in the SG (Additional file 5). There were no significant changes in *CAMK2A* gene expression with acute exercise (Fig. 3E), and no baseline differences between groups were detected (Additional file 2). In the untrained control group, only one DMP was detected, located on the *MYH2* promoter, with decreased methylation 3 h after resistance exercise (Additional file 5). Effect of acute exercise on skeletal muscle DNA methylation has been observed in other regions of the genome not covered by our panel [29, 49–55].

**Table 2:** Differentially methylated regions (DMRs) in response to exercise

***DMR: endurance group doing acute endurance exercise***							
**Associated gene**	**Associated feature**	**Length**	**nCpG**	**Pre DNA methylation**	**Post DNA methylation**	**Delta**	**areaStat**
*MYL3*	Promoter	118	6	60%	66%	6%	− 24
***DMRs: strength group doing acute resistance exercise***							
**Associated gene**	**Associated feature**	**Length**	**nCpG**	**pre (beta-value)**	**3 h (beta-value)**	**Delta**	**areaStat**
*MYL3*	Promoter	118	6	75%	70%	-5%	− 15
*CAMK2A*	Promoter	68	11	38%	40%	2%	− 22

Default parameters with a *p* value threshold of < 0.05 were utilised to identify DMRs. areaStat = sum of the test statistics of all CpG sites within a DMR (larger areaStat is more likely to be a DMR). See Additional File 5 for genomic locations of DMRs as well as information on differentially methylated positions (DMPs). Individual data values can be found in Additional file 6

### The influence of baseline methylation on the transcriptional response to exercise

Baseline differences in methylation could prime genes for more or less transcriptional activation in response to exercise [13, 26–28]. We therefore investigated if there were group differences in the transcritptional response to exercise that were associated with baseline differences in DNA methylation. The EG had higher intronic *FOXO3* methylation at baseline (Table 1) and was the only group with a significant increase in *FOXO3* expression after acute endurance exercise (Additional file 2). *CREB5* expression increased in both the SG and CG after resistance exercise but no changes were detected in response to endurance exercise (Additional file 2). Correspondingly, the EG had higher *CREB5* promoter methylation at baseline.

**Fig. 4:**
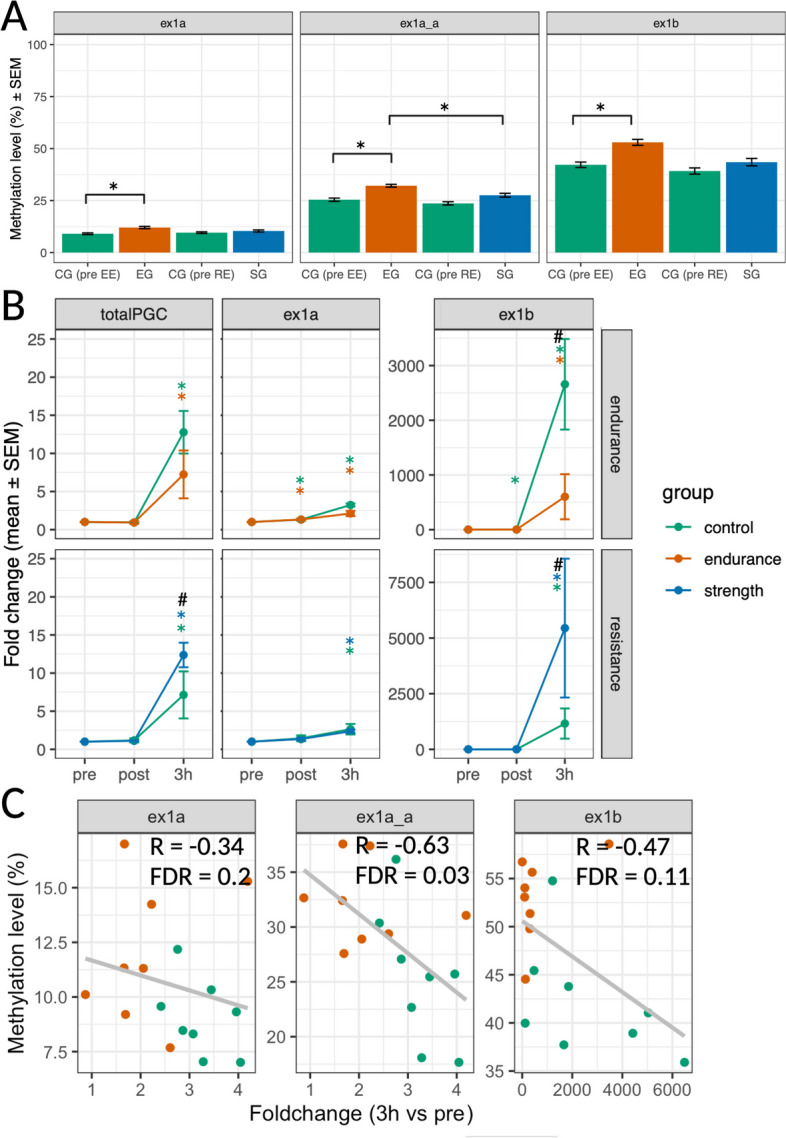
Endurance training modulates DNA methylation and transcriptional response of PGC1α. **A** Differentially methylated regions (DMR) of the PGC1α promoters at baseline (EG = endurance group, SG = strength group, CG = control group). ex1a and ex1a_a represent different DMRs on the proximal promoter, while ex1b a DMR on the alternative promoter. Results are shown as mean ± SEM for *n* = 8 subjects. ∗ indicates *p* < 0.05. **B** PGC1α expression assessed using RT-qPCR. TotalPGC = promoter unspecific transcripts. ex1a = transcripts from proximal promoter, ex1b = transcripts from alternative promoter. Results are shown as mean ± SEM for *n* = 8 subjects. Significance threshold *p* < 0.05: * = timepoint vs pre, # = EG or SG compared to CG. **C** Correlation between mean DMR methylation and fold change of associated promoter transcripts three hours after exercise. R = Spearman correlation coefficient. Grey line = linear regression model including subjects from the EG and CG doing endurance exercise. See Additional file 6 for individual methylation percentages and gene expression values

We were particularly interested in the Peroxisome proliferator-activated receptor gamma coactivator 1-alpha (*PGC-1α*), due to its known epigenetic regulation [29, 51, 56–58] and the differential regulation of its proximal and alternative promoters in response to exercise [59–63]. We did not observe exercise-induced changes in *PGC-1α* methylation in any group. However, we observed higher baseline methylation in the endurance athletes at the proximal (ex1a) and alternative (ex1b) promoters (Table 1, Fig. 4A). No difference in baseline transcription from the proximal or alternative promoters (assessed using qRT-PCR) was observed between groups (Fig. 4B). However, there were greater endurance exercise-induced expression changes from both promoters in the untrained controls compared to the endurance athletes (Fig. 4B), concordant with the lower promoter methylation observed in the untrained individuals. There was a moderate to strong negative correlation between the promoter-specific DMR methylation at baseline and the transcriptional response 3 h after endurance exercise (Fig. 4C). In response to resistance exercise, both the CG and SG demonstrated increased transcription from the proximal and alternative promoters, with the SG having a significantly higher fold change 3 h after exercise for total *PGC-1α* and the alternative promoter expression compared to the CG (Fig. 4B), despite no difference in baseline methylation. Baseline promoter-specific methylation levels and gene expression fold change 3 h after resistance exercise did not correlate significantly (ex1a: *R* = 0.46, FDR = 0.19; ex1a_a: *R* = 0.41, FDR = 0.19; ex1b =  − 0.14, FDR = 0.62).

## Discussion

In this study, we compared DNA methylation and associated transcription of exercise-responsive genes in skeletal muscle of well-characterised endurance- and strength-trained athletes with healthy, age-matched men, both at rest and after acute exercise. We found that exercise training background was associated with specific baseline DNA methylation differences in skeletal muscle, while one bout of exercise had little effect on DNA methylation in the investigated target genes.

Our investigation into the methylation patterns of the myosin heavy and light chain genes *MYH7*, *MYH6*, *MYL3*, and *MYH2* revealed group-distinct epigenetic signatures in skeletal muscle among endurance-trained, strength-trained, and untrained individuals that also correlated with VO_2_ peak. The observed methylation differences could be due to variations in muscle fiber type distribution between the groups, specifically the higher proportion of slow-twitch (type I) fibers in endurance-trained athletes as *MYH7* and *MYL3* methylation negatively correlated with type I fiber percentage. Our results align with previous findings of fiber type-specific methylation profiles in skeletal muscle of young healthy men, where slow-twitch fibers showed higher methylation of the *MYH2* promoter, introns and exons, while fast-twitch fibers showed higher intronic methylation of *MYH7*. Hypermethylation was associated with lower gene expression [64]. Hypomethylation of *MYH2*, *MYL3*, and *MYH7* has also been observed with increasing age, without differences in gene expression in human skeletal muscle [65]. These observations may be attributed to an increased abundance of type IIx muscle fibers, a reduced number of satellite cells, along with an infiltration of fat and immune cells in aged muscle [65]. Rodent muscle fibers extracted from either a slow or fast muscle continue to express the respective myosin chains in cell culture, even when other factors that influence fiber type, such as hormonal cues and neural stimulation, are eliminated [66], suggesting epigenetic factors contribute to fiber type-specific transcriptional regulation. Human satellite cells simultaneously express both slow and fast myosin chains in culture [67], allowing fusion to any fiber type. Understanding the influence of methylation on skeletal muscle fiber type could provide valuable insights for future therapeutic strategies. For example, in the early stages of Duchenne muscular dystrophy, type I fibers are less affected compared to type II fibers, and it has been suggested that symptoms and disease progression could potentially be attenuated by inducing more type I muscle fibers [68]. Other muscular dystrophies, sarcopenia, and aging are also associated with changes in fiber type distribution [69–71]. Slow-twitch muscle fibers possess superior quality control mechanisms with more active protein synthesis and turnover rates, which may promote resilience to age-related sarcopenia [71].

Differences in DNA methylation between groups were also present in several transcription factors. For the MRFs, we observed mixed results; some showed hypermethylation in the EG and SG compared to the CG, while *MYF5* for example was hypomethylated in the SG compared to the EG and CG. The increased methylation state of MRFs in endurance trained athletes is consistent with previous data showing hypermethylation of MRFs and *MEF2* in response to a 3-month endurance training intervention in skeletal muscle [24]. The same *MEF2A* enhancer that showed increased methylation in endurance-trained men in our study was reported to decrease in methylation with age, whereas the *MYF5*, *MyoD*, and *MYOG* promoters showed increased methylation with age [65]. *MYOG* methylation correlated with VO_2_ peak and was identified as an upstream regulator of DEGs in our endurance trained athletes. While MYOD1 was another top-ranked transcription factor influencing DEGs in the endurance group, its methylation positively correlated to leg strength. A recent study on DNA methylation of the myogenic regulatory factors *MYOD1*, *MYF5*, and *MYF6* showed reduced methylation at 4 h and 8 h after exercise, accompanied by increased expression of *MYOD1* and *MYF6*, although changes were small [55]. In contrast, an earlier study found that *MYOD1* and *MEF2A* methylation remained stable after acute endurance exercise [29]. Similarly, we did not observe any exercise-induced changes in methylation of *MRFs* in our study. In summary, the methylation patterns of MRFs appear to be affected by training background. However, given the absence of corresponding changes in our gene expression data, the functional implications of these methylation patterns remain uncertain.

We found higher DNA methylation of intronic regions of *FOXO1* and *FOXO3* in endurance trained athletes that correlated with aerobic capacity. Hypermethylation of *FOXO1* and *FOXO3* enhancers located within our identified DMRs has also been shown in response to 3 months of endurance training in human skeletal muscle [24]. While all groups showed a significant increase of *FOXO1* expression after exercise, *FOXO3* only increased in endurance trained athletes. The *FOXO3* DMR has previously been reported as an enhancer [24], which are known to display dynamic methylation [72]. Notably, in cancer, the methylation levels of enhancers have a closer association with gene expression changes than promoter methylation [73, 74]. It has been reported that environmental stimuli, such as exercise, may primarily impact enhancers and intragenic regions. For instance, the predominant methylation differences related to child maltreatment are found in intragenic regions [75]. Exercise training and cardiorespiratory fitness has been shown to affect DNA methylation changes in enhancers, gene bodies, and intergenic regions, with lesser alterations in promoter regions [24, 25]. Aging-related DNA methylation changes in skeletal muscle were concentrated around active transcription start sites and enhancers [65]. While promoter methylation generally results in gene silencing by blocking the access of RNA polymerase [12], the effects of intragenic methylation on gene transcription are highly variable [13, 24, 65, 76]. Gene body methylation has for example been associated with highly expressed genes throughout the human genome [77].

*FOXO3* and *FOXO1* are important for cellular homeostasis and for response to exercise-induced metabolic stress by optimising protein turnover during recovery [31]. They are known for their cytoprotective effect and as potential targets to combat muscle aging [78]. *FOXO1* and *FOXO3* are hypomethylated with age in an epigenome-wide meta-analysis of human skeletal muscle [65]. DNA methylation and expression of *FOXO3* differs between male and female skeletal muscle, which may be explained by differences in muscle fiber type proportions [79]. Taken together, we suggest that the detected intragenic *FOXO3* DMR in endurance-trained men, possibly due to higher proportion of type I fibers in this group, influences exercise-induced *FOXO3* expression and thereby contributes to endurance adaptation. The observed methylation and expression patterns of *FOXO3* could result in improved coping with metabolic disruption after acute exercise and potentially influence muscle aging.

*CREB5* exhibited higher methylation for an intronic DMR in the EG compared to the CG, while there was no difference in methylation between the CG and SG. Only resistance exercise increased *CREB5* transcription. Previous research has reported higher methylation of *CREB5* in female compared to male myoblasts and myotubes, which was associated with lower expression levels [80]. We observed a moderate negative correlation between *CREB5* gene expression and methylation. This suggests that long-term endurance training may increase *CREB5* methylation, resulting in lower *CREB5* induction following acute endurance exercise. Similar effects of baseline methylation levels on exercise-induced transcription were observed for *FOXO3*, suggestive of a ‘primed’ gene regulatory state. Methylation changes induced by an acute hypertrophic stimulus in mice has been suggested to represent a ‘primed’ state, preparing the muscle cells to respond to stimulation more efficiently [26]. In trained muscle, altered DNA methylation patterns of regulatory genes in particular may affect expression after one bout of exercise [81]. After an initial resistance exercise loading phase, epigenetic changes persisted through subsequent unloading and reloading periods in humans, suggesting the presence of a resistance training-induced epigenetic memory [53, 54]. This eventually resulted in further upregulation of a subset of genes with retained methylation states when reloading occurred. Our results support the idea that training alters DNA methylation of selected genes which can influence the transcriptional response to exercise in trained individuals.

Elevated skeletal muscle *PGC-1α* promoter methylation is associated with type-II diabetes and inactivity [51, 57]. Previous studies have shown exercise-induced demethylation of the proximal *PGC-1α* promoter as well as after long-term electrical stimulation in patients with spinal cord injuries [29, 56]. We did not detect any exercise-induced changes in *PGC-1α* methylation; however, there were training background-dependent baseline methylation differences in both the proximal and alternative promoter as well as different degrees of transcriptional activation after acute exercise. PGC-1α methylation at baseline correlated with VO_2_ peak but not with type I fiber percentage. Baseline *PGC-1α* expression did not differ between groups, but the endurance group showed attenuated exercise-induced increase in proximal and alternative promoter transcription compared to the control group. To our knowledge, this is the first study to investigate the methylation level of the proximal and alternative promoters in human skeletal muscle in response to exercise. Our transcriptional findings align with previous data demonstrating that only *PGC-1α* transcripts from the alternative promoter changed after exercise in endurance-trained athletes [62]. Transcription from the proximal promoter following one-legged knee-extension exercise was blunted after 6 weeks of endurance training [82]. While endurance exercise intensities between trained and untrained men were matched in relative intensity in our study, fiber type differences could explain the transcriptional response variability. Untrained men had a higher proportion of type II fibers, which are characterised by a pronounced metabolic shift during exercise and exhibit a higher *PGC-1α* transcriptional response to endurance exercise compared to type I fibers [83, 84]. In addition to potential differences in metabolic perturbation for different fiber types, higher *PGC-1α* methylation in trained men could also blunt the exercise-induced changes in *PGC-1α* expression.

Our study has several limitations. An important consideration when analysing DNA methylation in bulk tissues is cellular heterogeneity and the associated cell type-specific epigenetic marks [26, 85]. Given that autosomes are diploid, making a CpG dinucleotide either 0%, 50%, or 100% methylated, observing slight methylation variations suggests that only a minor subset of cells undergo differential methylation at specific sites. This could considerably affect the respective cells functionality. Skeletal muscle tissue includes, e.g. muscle fibers, immune and endothelial cells, erythrocytes, and fibroblasts [86]. Although we found minimal differences in cell type composition between groups, some differences especially in fiber type distribution likely influenced our results and could explain the lack of significant methylation shifts following exercise in the selected target genes covered by our study. Our sample size was relatively small, which limits the statistical power to detect changes in DNA methylation and increases the risk of type II errors, particularly for small effect sizes seen for methylation changes in response to environmental stimuli [21, 55]. While the magnitude of DNA methylation differences in our study is similar to what has been observed in other studies on exposure to environmental stimuli [21, 55, 87, 88], small effect sizes also limit power. Furthermore, the relationship between DNA methylation and gene expression is complex and multifaceted. Our findings should be interpreted within the vast landscape of gene regulation, where subtle shifts in methylation can play a role in a broader network of regulatory interactions, depending on the genomic context, cell type, and transcription factor activity. Furthermore, we measured DNA methylation at two time points after exercise and therefore may have missed transient changes occurring at other time points. DNA methylation is influenced by various intrinsic and extrinsic factors, including diet, age, genetic background, stress, and other lifestyle factors [19]. Although a recent study reported no sex differences in the skeletal muscle methylome with lifelong training [25], there are significant differences between male and female skeletal muscle and future studies should also include women [89]. We controlled for some of the extrinsic variables by ensuring all participants were rested and consumed a standardised breakfast on the intervention days, but multiple confounders remain, including individual genetic differences. To test for stochastic or technical factors that could influence the measured methylation levels, we compared two baseline samples from each subject in the control group, one before endurance and one before resistance exercise. The absence of significant methylation differences between the two baseline samples indicates that the differences between groups are not due to sample handling or other environmental triggers. Moreover, prior transcriptomic analyses of the two baseline samples from the same subjects found no differentially expressed genes [11].

A comprehensive understanding of exercise-induced epigenetic changes and their influence on the health benefits associated with long-term training is crucial from a basic human biology perspective and carries therapeutic potential. By deciphering the DNA methylation signature of trained muscle, we could potentially develop drugs that emulate the benefits of exercise for those unable to participate in physical activities and retain muscle function in later life. Future studies should explore the precise molecular pathways governing exercise-induced epigenetic changes in skeletal muscle and their broader implications for health and human performance.

## Conclusions

This study highlights differences in DNA methylation between trained and untrained men for genes that are central to exercise adaptation. We propose that skeletal muscle fiber type and DNA methylation patterns are closely intertwined. Furthermore, we suggest that the training-induced baseline DNA methylation landscape in skeletal muscle influences the transcriptional response of certain genes to exercise in a training-background dependent manner.

## Methods

### Subject characteristics

Twenty-four healthy men between 35 and 52 years (41.6 ± 6) of age were recruited into one of three study groups: (1) endurance-trained athletes (EG, *n* = 8), (2) resistance-trained athletes (SG, *n* = 8), or (3) untrained control subjects (CG, *n* = 8). Subjects were selected based on a questionnaire addressing their training habits and physiological testing of their endurance capacity (VO_2_ peak) and their maximal quadriceps torque using Biodex. The endurance trained athletes reported at least 15 years of endurance training in the form of running, biking, or a combination of both at a high level (more than 3 times per week). The strength-trained athletes were involved in heavy strength training regimes for the past 15 years (powerlifting and Olympic lifting). The transcriptional and metabolic profiles of the skeletal muscle for these individuals have been analysed in detail elsewhere [11].

### Intervention

Control subjects performed one endurance exercise bout (EE) and one resistance exercise bout (RE) with 4–8 weeks of wash-out in-between the two interventions. Endurance-trained subjects performed EE and strength-trained athletes RE. All subjects were instructed to eat a standardised breakfast three hours before the exercise bout. Prior to the intervention day, they were asked not to exercise for 72 h. Only water was allowed to be consumed during the intervention and until the last sampling timepoint. All interventions started between 8:00 and 9:00 am to control for circadian rhythm effects. The RE protocol consisted of nine sets of eight repetitions on a knee extension machine (set length 40 s; set break 150 s) at 80% of the respective measured one repetition maximum. The EE protocol consisted of 30 min of cycling at 75% of the measured Wpeak (assessed during the VO_2_ peak test). Subjects were pushed to exhaustion during the sessions. Skeletal muscle biopsies were obtained from *M. vastus lateralis* at rest before the exercise, immediately after, and 3 h after the exercise bouts using the Bergstrom needle technique [90]. To minimise the potential effects of local inflammation, the incisions for the second biopsy in each leg were made at least 2 cm proximal to the previous biopsy. Muscle samples were immediately frozen in liquid nitrogen and stored at – 80 °C until analysis.

### Genomic DNA extraction

Five to ten mg of muscle was homogenised in Cell Lysis Solution using a bead homogeniser. DNA was isolated from all samples using the Gentra Puregene Tissue Kit (Cat. No. 158063) according to the manufacturer’s instructions. The final DNA concentration was measured with absorbance at 260/280 nm using NanoDrop 2000 (Thermo Scientific). All samples had OD 260/280 of 1.8–2.0.

### Enzymatic methyl sequencing

For the design of the targeted methylation panel, we synthesised information from a variety of sources, including the chromatin states in skeletal muscle obtained from the Roadmap Epigenomics Project [91], the most recent data from the GeneHancer database [92], and the Eukaryotic Promoter Database [93]. Our focus for the panel was on regulatory regions of genes known to play a role in the acute response to exercise as well as training adaptations. Specifically, to identify promoter regions, we utilised the Eukaryotic Promoter Database to pinpoint the transcription start sites (TSS) for specific genes. Subsequently, probes were designed to encompass an area that, at a minimum, extends 500 bp upstream of the TSS and includes the adjacent downstream base pairs. For the identification of enhancer-associated regions, we relied on the GeneHancer database, combined with chromatin state data from the Roadmap Epigenomics Project. Moreover, some enhancers were sourced from the analysis of the epigenome in human skeletal muscle after training [24]. The final design consisted of 57 target regions across 37 different genes. The custom targeted methylation panel was synthesised in collaboration with Twist Bioscience (https://www.twistbioscience.com/). The total size was 66,588 bp covered by 561 probes at a length of 120 bp/probe. Fourteen probes were removed due to repeats resulting in a final design size of 64,974 bp with 97.55% coverage of our included targets.

A total of 500 ng DNA, dissolved in 50 μl of EB buffer, was sent to the SNP&SEQ Technology Platform in Uppsala (Sweden) for quality assessment, library preparation, sequencing, and sequence alignment. To ensure adequate amount and quality of DNA, the quality of the samples was assessed using the Agilent TapeStation system (Agilent Technologies), while the concentration was determined using the Invitrogen™ Qubit/Quant-iT assay (ThermoFisher Scientific). All samples passed quality control. Sequencing libraries were generated from 200 ng of DNA using the NEBNext Enzymatic Methyl-seq sample preparation kit, specifically the Twist NGS Methylation Detection System (catalogue numbers 101977, 101,978, and 101,979). Unique dual indexes were employed from Twist Bioscience and New England Biolabs. The entire process of library preparation was carried out according to the guidelines provided by the manufacturers. The prepared libraries, ranging from 160 to 187.5 ng, were then hybridised using a Twist Methy Custom Panel probe panel from Twist Bioscience. Paired-end sequencing with a read length of 150 base pairs was performed on the NovaSeq 6000 system, utilising an SP flowcell and version 1.5 sequencing chemistry. EM-seq minimises DNA damage during methylation analysis and produces superior resolution, mapping rate, and GC content representation compared to traditional bisulfite conversion methods [94–97].

### Targeted EM-seq data processing and quality control

Raw sequencing data in FastQ format was pre-processed (removal of adapter contamination, trimming of low-quality regions), and the reads were aligned to the hg38 human genome reference using the nf-core/methylseq pipeline (v1.6.1) [44]. Before performing the alignment step with Bismark (v0.23.0), all reads were trimmed using TrimmGalore (v0.6.6). The pipeline included extensive quality control of pre- and post-mapping steps (MultiQC; v1.10.1). Alignments with identical mapping positions were removed to avoid technical duplication in the results (Bismark Deduplication; v0.23.0) before cytosine methylation calls were extracted (Bismark methXtract; v0.23.0). For downstream analysis, only CpG sites in the target panel regions with 10X coverage were used.

### RNA extraction and sequencing

Total RNA from skeletal muscle was extracted using the phenol-based TRIzol method, and quantity and quality of RNA was checked by using the 2100 Bioanalyzer System (Agilent Technologies, Santa Clara, CA, USA). Libraries underwent preparation through poly-A selection (utilising TruSeq mRNA kits by Illumina, located in San Diego, CA, USA) and were multiplexed at the National Genomics Infrastructure Sweden. The cBot system was utilised for clustering, and sequencing was carried out using the NovaSeq6000 system (operating with NovaSeq Control Software 1.6.0 and RTA v3.4.4), employing a dual-lane configuration with a 2 × 151 cycle in the NovaSeqXp workflow, utilising an ‘S4’ mode flow cell. Conversion from Bcl to FastQ format was accomplished using version 2.20.0.422 of bcl2fastq, part of the CASAVA suite. The Sanger/phred33/Illumina 1.8 + quality scale was used as quality scale. Quality control and analysis were conducted using the nfcore/rnaseq pipeline [44].

### cDNA synthesis and real-time quantitative PCR

For real-time qPCR, 200 ng RNA was reversed transcribed to single-stranded cDNA using MultiScribeTM Reverse Transcriptase and random primers (Applied Biosystems, Foster City, CA) in a total volume of 20 μl. PGC-1α mRNA was quantified with real-time RT-PCR [total PGC-1α, PGC-1α -ex1a, PGC-1α-ex1b, trunc-PGC-1α, and nontrunc-PGC-1α]; see Ydfors et al. [60] for primer design description and efficiency testing. Primers were synthesised by Cybergene AB. Stockholm, Sweden (see Additional file 1: Table S4 for primer sequences). Five microliters of cDNA sample, forward primer (final concentration 0.4 μM), reverse primer (final concentration 0.4 μM), and SYBR Green PCR Master Mix (Applied Biosystems) were added up to a total reaction volume of 10 μl per sample. Beta-2-microglobulin (*BTM*) [98], TATA box-binding protein (*TBP*), and cyclophilin A (*PPIA*) [99] were used as stable endogenous controls across all timepoints. All samples were run in duplicates in the Applied Biosystems 7500 Fast Real-Time PCR System.

### Statistical analysis

Differential methylation analysis was performed using the DSS package in R (4.3.0) [100, 101]. Testing for differential methylation was performed using a Wald test based on a beta-binomial distribution model, where the test statistics consider both biological variation among replicates and sequencing depth. Biological variation is characterised by the dispersion parameter, which was estimated using a shrinkage estimator based on a Bayesian hierarchical model. A *p*-value of < 0.05 was used to call DMRs and DMPs. According to the default settings of the DSS package, each DMR consisted of at least three CpG sites. To compare timepoints within a group, as well as to compare endurance and strength exercise within the control group, a fixed-effect model was used to accommodate the paired design. For visualisation purposes, sets of DMRs from different comparisons were plotted together if they shared at least one CpG site. Gene and functional annotation was done with the TxDb.Hsapiens.UCSC.hg38.knownGene package in R [102]. Information from several sources, including the chromatin states in skeletal muscle derived from the Roadmap Epigenomics Project [91], the latest data from the GeneHancer database [92], and the Eukaryotic Promoter Database [93], were incorporated for functional annotation of DMRs. Principal component analysis (PCA) of methylation percentages was conducted by applying R base (4.3.0) functions and the factoextra package (1.0.7) [103].

The relative expression level of each PGC-1α isoform at rest was determined using the 2^−∆∆Ct^ method [104]. Since the criteria for normal distribution were not met, a Kruskal–Wallis test was used to identify differences in baseline expression and fold change among the three groups, followed by a Dunn’s post hoc test. A Friedman one-way analysis of variance followed by a post hoc Wilcoxon matched pairs signed-rank test was used to assess fold-change differences within each group performing resistance or endurance exercise. Given the multiple comparisons, *p*-values were adjusted employing the Benjamini–Hochberg procedure [105]. Spearman correlation was used to assess the relationship between methylation percentage and gene expression, fold change of PGC-1α transcripts, fiber type, VO_2_ peak, and leg strength. For transcriptional regulatory analysis using Lisa, we took the up- and down-regulated DEG lists (FDR < 0.05) of the baseline control versus endurance group comparison as input [45]. Cell type composition analysis was performed using CIBERSORTx for digital immune cell deconvolution with the curated signature matrix LM22 signature matrix, as previously described [48]. The CIBERSORTx output was further analysed by Kruskal–Wallis and post hoc Dunn-test with Benjamini–Hochberg correction for multiple testing.

### Supplementary Information

Additional file 1: Table S1. (Targeted DNA methylation panel). Table S2. (Proportions of different immune cells estimated by CIBERSORTx). Table S3. Correlations between methylation percentage at baseline and A) Gene expression, B) VO2 peak, C) Leg Strength and D) Type I Fiber percentage). Table S4. (Primer sequences).

Additional file 2. Gene expression data.

Additional file 3. Epigenetic Landscape In Silico Deletion Analysis (Lisa).

Additional file 4. Differential Methylation Analysis between groups.

Additional file 5. Differential Methylation Analysis within groups between timepoints.

Additional file 6. Individual data points for Tables and Figures.

## Data Availability

All data generated or analysed during this study are included in this published article, its supplementary information files, and the European Genome-phenome Archive (RNAseq and and EMseq data: https://ega-archive.org/studies/EGAS00001006139).

## Abbreviations

*bp*: Base pair
*BTM*: Beta-2-microglobulin
*CAMK2A*: Calcium/calmodulin dependent protein kinase 2 alpha
CG: Control group
CpG: Cytosine-phosphate-guanine
*CREB*: CAMP response element-binding protein
CREB1: CAMP response element-binding protein 1
*CREB5*: CAMP responsive element-binding protein 5
*CS*: Citrate synthase
*DCN*: Decorin
*CFD*: Complement factor D
DEGs: Differentially expressed genes
DMPs: Differentially methylated positions
DMRs: Differentially methylated regions
DNMT3A: DNA methyltransferase 3 alpha
EE: Acute endurance exercise bout
EG: Endurance group
EM-seq: Enzymatic methyl sequencing
*ESAM*: Endothelial cell adhesion molecule
ex1a: Exon 1a, *PGC-1α* proximal promoter
ex1b: Exon 1b, *PGC-1α* alternative promoter
FDR: False discovery rate
FOXO: Forkhead box O
FOXO1: Forkhead box O1
*FOXO3*: Forkhead box O3
*GSN*: Gelsolin
HIF1A: Hypoxia inducible factor 1 subunit alpha
*ICAM1*: Intercellular adhesion molecule 1
*KDR*: Kinase insert domain receptor
Lisa: Epigenetic Landscape In Silico deletion Analysis
MAPK12: Mitogen-activated protein kinase 12
*MEF2A*: Myocyte enhancer factor 2
MRFs: Myogenic regulatory factors
*MSTN*: Myostatin
*MTOR*: Mechanistic target of rapamycin kinase
*MYC*: MYC proto-oncogene
MYF5: Myogenic factor 5
MYF6: Myogenic factor 6
*MYH2*: Myosin heavy chain 2
*MYH7*: Myosin heavy chain 7
*MYH6*: Myosin heavy chain 6
*MYL3*: Myosin light chain 3
*MYOD1*: Myogenic differentiation 1
*MYOG*: Myogenin
*NDUFA4*: NADH dehydrogenase (ubiquinone) 1 alpha subcomplex 4
Nm: Newton metres
NK cells: Natural killer cells
*NRF1*: Nuclear respiratory factor 1
*PAX7*: Paired box 7
PC: Principal component
*PDK4*: Pyruvate dehydrogenase kinase 4
*PECAM*: Platelet endothelial cell adhesion molecule 1
*PGC-1α*: PPARG coactivator 1 alpha *PPARGC1A*
*PPIA*: Cyclophilin A
QC: Quality control
qRT-PCR: Quantitative reverse transcription-polymerase chain reaction
RE: Acute resistance exercise bout
SD: Standard deviation
SG: Strength group
*SMAD3*: SMAD family member 3
*TBP*: TATA box-binding protein
*TEK*: TEK receptor tyrosine kinase
*TET2*: Tet methylcytosine dioxygenase 2
*TET3*: Tet methylcytosine dioxygenase 3
*TFAM*: Mitochondrial transcription factor A
TSS: Transcription start sites
VO_2_ peak: Peak oxygen uptake
*VEGFA*: Vascular endothelial growth factor A
*VWF*: Von Willebrand factor
