# Supplementary material for: DNA methylation of exercise-responsive genes differs between trained and untrained men
Source: BMC Biol. 2024 Jul 4;22:147. doi: 10.1186/s12915-024-01938-6 (PMC11225400; doi:10.1186/s12915-024-01938-6)
Supplement: Supplementary file 1 — Additional file 1: Table S1. (Targeted DNA methylation panel). Table S2. (Proportions of different immune cells estimated by CIBERSORTx). Table S3. Correlations between methylation percentage at baseline and A) Gene expression, B) VO2 peak, C) Leg Strength and D) Type I Fiber percentage). Table S4. (Primer sequences). [file 12915_2024_1938_MOESM1_ESM.pdf]

Supplementary Table 1: Targeted DNA methylation panel. Genomic coordinates are based on genome assembly GRCh38/hg38.

Target region	Chromosome	Start	End	Length
ATF2 Promoter	chr2	175181611	175182358	747
CAMK2A Enhancer	chr5	150256130	150256868	738
CAMK2A Promoter	chr5	150289630	150290353	723
CAMKIV Promoter	chr5	111223579	111224486	907
CREB1 Promoter	chr2	207529048	207530297	1249
CREB5 Promoter	chr7	28685390	28686760	1370
CREB5 TSS	chr7	28412446	28412669	223
CS Promoter	chr12	56299357	56301150	1793
ESRRA Promoter	chr11	64305030	64306212	1182
FOXO1 Enhancer	chr13	40598208	40599640	1432
FOXO1 Promoter	chr13	40666208	40667577	1369
FOXO3 Enhancer	chr6	108650156	108651861	1705
FOXO3 Promoter	chr6	108558752	108560683	1931
HES1 Promoter	chr3	194135518	194136612	1094
HIF1A Promoter	chr14	61694712	61695621	909
HIF1A Promoter/Enhancer	chr14	61697314	61698034	720
MAPK12 Enhancer	chr22	50248041	50249380	1339
MAPK12 Promoter	chr22	50261277	50262808	1531
MEF2A Enhancer	chr15	99718988	99719684	696
MEF2A Promoter	chr15	99565358	99566091	733
MSTN Promoter/Enhancer	chr2	190062542	190066015	3473
MTOR Enhancer	chr1	11157965	11158698	733
MTOR Promoter	chr1	11261784	11262982	1198
MYC Enhancer	chr8	127898412	127898532	120
MYF5 Promoter	chr12	80716415	80717349	934
MYF6 Promoter	chr12	80706525	80707694	1169
MYH2 Promoter	chr17	10549558	10550157	599
MYH6 Promoter	chr14	23409253	23409513	260
MYH7 Promoter	chr14	23435560	23436160	600
MYL3 Promoter	chr3	46863344	46863943	599
MYOD1 Core Enhancer	chr11	17699561	17699898	337
MYOD1 DRR Enhancer	chr11	17713956	17715330	1374
MYOD1 Promoter	chr11	17718561	17720213	1652
MYOG Promoter	chr1	203085593	203087076	1483
NDUFA4L2_1 Promoter	chr12	57236323	57237603	1280
NDUFA4L2_2&3 Promoter	chr12	57240621	57241360	739
NRF1 Promoter	chr7	129610993	129611946	953
PAX7 Promoter	chr1	18630893	18631726	833
PDK4 Enhancer	chr7	95592725	95592845	120
PDK4 Promoter	chr7	95596415	95597014	599

PPARGC1A alternative Promoter	chr4	23903795	23904977	1182
PPARGC1A Enhancer?	chr4	23880612	23883359	2747
PPARGC1A Enhancer?	chr4	23934209	23935339	1130
PPARGC1A Enhancer?	chr4	23945150	23946601	1451
PPARGC1A proximal Promoter	chr4	23887877	23891221	3344
PRKAA1 Promoter	chr5	40797940	40798913	973
PRKAA2 Promoter	chr1	56644815	56645414	599
SLC2A4 Promoter	chr17	7279539	7283489	3950
SMAD Enhancer	chr15	67165245	67166183	938
SMAD3 Enhancer	chr15	67098033	67098808	775
SMAD3 Enhancer	chr15	67136625	67136886	261
SMAD3 Promoter	chr15	67065343	67065942	599
TBP promoter	chr6	170553777	170554471	694
TFAM Promoter	chr10	58384572	58385550	978
TGFB1 Promoter	chr19	41353376	41354375	999
VEGFA Enhancer	chr6	44057596	44058015	419
VEGFA Promoter	chr6	43769299	43772782	3483

Supplementary Table 2: Proportions of different immune cells estimated by CIBERSORTx (see Methods for details).

CellType	CG	EG	SG	Significant difference
B.cells.memory	0,00085012	0	0,0013107	
B.cells.naive	0,13336205	0,12519107	0,11428227	
Dendritic.cells.activated	0,00637968	0,00181708	0,00585571	
Dendritic.cells.resting	0,00122349	0,00085793	0,00574504	
Eosinophils	0,02522647	0,01423789	0,04422174	
Macrophages.M0	0,00684289	0,00112732	0	
Macrophages.M1	0,06982851	0,05455127	0,06964833	
Macrophages.M2	0,46896146	0,37532914	0,5090656	* EGvsSG
Mast.cells.activated	0,01099842	0,00549446	0,00299834	
Mast.cells.resting	0,26890452	0,24829314	0,27521446	
Monocytes	0,06148975	0,05800037	0,07526069	
Neutrophils	0,06795992	0,01822721	0,03432295	
NK.cells.activated	0,06457682	0,05828663	0,0624323	
NK.cells.resting	0,09553358	0,16741426	0,11907675	*EGvsCG
Plasma.cells	0,05870218	0,01477505	0,01686137	
T.cells.CD4.memory.activated	5,6021E-05	0	0	
T.cells.CD4.memory.resting	0,29569428	0,30169222	0,37713086	
T.cells.CD4.naive	0,00340222	0,01025579	0,01476943	
T.cells.CD8	0,05837374	0,03959892	0,01652016	
T.cells.follicular.helper	0,07121601	0,07461673	0,10550473	
T.cells.gamma.delta	0	0	0	

T.cells.regulatory..Tregs.	0,00217911	0,0010491	0,00491188	

CG = control group, EG = endurance group, SG = strength group

Supplementary Table 3: Correlations between methylation percentage at baseline and A) Gene expression, B) VO_2peak, C) Leg Strength and D) Type I Fiber percentage

	A) Gene expression		B) VO_2peak		C) Leg Strength		D) Type I Fiber %	
Gene	R	FDR	R	FDR	R	FDR	R	FDR
CREB1	0.21	0.43	-0.01	0.94	-0.26	0.66	0.49	0.04
CREB5	-0.38	0.11	0.59	<0.01	-0.10	0.84	0.26	0.31
FOXO1	-0.25	0.36	0.70	<0.01	0.07	0.89	0.46	0.06
FOXO3	-0.30	0.22	0.71	<0.01	-0.12	0.84	0.63	0.01
HIF1A	0.17	0.50	-0.18	0.35	-0.30	0.66	0.30	0.25
MAPK12	-0.39	0.11	0.69	<0.01	0.04	0.89	0.31	0.24
MEF2A	-0.14	0.59	0.23	0.23	-0.19	0.70	0.20	0.42
MTOR	-0.09	0.70	0.43	0.02	-0.04	0.89	0.51	0.05
MYF5	-0.23	0.39	0.32	0.09	-0.27	0.66	0.50	0.04
MYH2	-0.39	0.11	0.54	<0.01	-0.02	0.89	0.09	0.77
MYH7	-0.18	0.50	-0.45	0.02	-0.24	0.66	-0.62	<0.01
MYL3	-0.52	0.04	-0.51	0.01	-0.05	0.89	-0.83	<0.01
MYOD1	0.12	0.62	0.33	0.08	0.66	<0.01	0.001	0.99
MYOG	0.01	0.98	0.42	0.02	0.10	0.84	0.002	0.99
PPARGC1A	-0.43	0.11	0.64	<0.01	0.10	0.84	0.24	0.33
SMAD3	-0.37	0.11	0.45	0.02	-0.14	0.84	0.34	0.20

Supplementary Table 4: Primer Sequences

Amplicon Name	Forward Primer	Reverse Primer
Total PGC-1α	GTGGTGCAGTGACCAATGAG	CTGCTAGCAAGTTTGCCTCA
PGC-1α -ex1a	TGTATGGAGTGACATCGAGTGT	GCTGGTCTTCACCAACCAGA
PGC-1α -ex1b	GACACACATGTTGGGGTTATCA	ACCAACCAGAGCAGCACATTT
B2M	AAGGACTGGTCTTTCTATCTC	GATCCCACTTAACTATCTTGG
TBP	TGTGCTCACCCACCAACAAT	TCTGCTCTGACTTTAGCACCTG
PPIA	ATGGTTCCCAGTTTTTCATC	CTCCACAATATTCATGCCTTC
